# Control of biofilm-producing *Pseudomonas aeruginosa* isolated from dairy farm using Virokill silver nano-based disinfectant as an alternative approach

**DOI:** 10.1038/s41598-022-13619-x

**Published:** 2022-06-08

**Authors:** Sahar Abdel Aleem Abdel Aziz, Rehab Mahmoud, Manar Bahaa El Din Mohamed

**Affiliations:** 1 Department of Hygiene, Zoonoses and Epidemiology, Faculty of Veterinary Medicine, Beni-Suef University, Beni-Suef, 62511 Egypt; 2 Department of Chemistry, Faculty of Science, Beni-Suef University, Beni-Suef, 62511 Egypt

**Keywords:** Biochemistry, Microbiology, Molecular biology, Environmental sciences

## Abstract

*Pseudomonas aeruginosa* (*P. aeruginosa*) is an important opportunistic pathogen that is responsible for many clinical infections in both animals and humans. This study aimed to detect the prevalence of *P. aeruginosa* in dairy farm's that possess a great importance to dairy industry where it shares in milk spoilage. Evaluation of the efficacy of commonly used disinfectants to control the pathogen in dairy environment and finding a way to overcome high resistance to the used agents. Samples (*n* = 250) were collected from different environmental components, milk, and milkers' hands. Pathogens were isolated, biofilm was detected and their sensitivity against two commonly used disinfectants and against silver nanoparticles and Virokill AgNPs at different concentrations and contact times were tested. The pathogen significantly prevailed in milk samples (70.0%, *P* < 0.001). 50 out 74 isolates were biofilm-forming that was significantly obtained from environment (71.8%, P < 0.001). *P*. *aeruginosa* showed variable degree of resistance to tested disinfectants but it was significantly sensitive to Virokill AgNPs (200/1000) mg/l at exposure time 24 h (*P* < 0.001). It was concluded that using Virokill AgNPs in regular sanitation and disinfection of dairy farms, this helps the control of *P. aeruginosa* subsequently increasing milk quality and improving dairy industry and protecting human health.

## Introduction

*Pseudomonas* spp. is a ubiquitous pathogen that is commonly found in dairy farms' environment^1,2^. *P. aeruginosa* is a Gram-negative motile bacterium that is associated with various diseases in both humans and animals. In humans it is responsible for many infections such as pneumonia, septicemia, and necrosis in immunocompromised individuals^3^, meanwhile in animals it causes dermatitis, otitis, and urinary tract infection^4^. It also has been associated with many cases of clinical and subclinical mastitis in dairy ruminants^5,6^. Due to its low nutritional requirement and its ability to form a biofilm, *Pseudomonas* sp. were able to survive in different environments and they were allowed to grow and survive on equipment and utensils used in dairy production such as bulk milk tank, milking machines, pipelines, soiled bedding, humid soil, in air and water; subsequently increasing the risk of spreading infections to other animals and humans^7–9^ and very much for the same reasons the World Health Organization has listed it as a critical priority pathogen^10^.

*P. aeruginosa* possess several and variable virulence factors and antimicrobial determinants that are harbored in its genome, these factors provide the pathogen with metabolic flexibility and the ability to adapt adverse conditions^11^. For instance polymerized flagellin (*fliC*) gene is not only responsible for microbial motility but also responsible for binding to the membrane glycolipid asialo-GM1 on the apical surface of the lung epithelial cells^12^. Besides Flagellar attachment provided by *fliC* it helps in initial biofilm establishment, where motility allows cell dispersal^13^.

Additionally, the exotoxin A encoded *tox A* gene play a potential role in of *P. aeruginosa* pathogenicity where it is responsible for its cytotoxic capacity. It is a protein involved in pro-inflammatory cytokine synthesis stimulation. These virulence factors are mainly cooperated during *P. aeruginosa* colonization of the host cell and biofilm formation, allowing for further infection, and increasing bacterial pathogenicity^14^.

Biofilm formation is a microbial feature that increases' the bacterial resistance to antimicrobial agents (both antibiotics and disinfectants), biofilm is difficult to remove also it consider as a source of contamination to dairy products and subsequently human consumers with such biofilm producing pathogens^15,16^. *Pseudomonas* spp. is one of biofilm producing microorganisms. *Pseudomonas*'s antimicrobial resistance is adapted by the formation of biofilms on the contaminated surfaces that act as a diffusion barrier that limit the access of these agents to the bacterial cells and hinder their action^17^.

Providing hygienic measures during milking process and using disinfectants to keep the environment surrounding dairy animals clean is an important measure to reduce the contamination with such pathogen^18^. Despite the availability of many commercial disinfectants used in the cleaning and sanitation process in dairy farms, they show weak efficacy to limit contamination of both raw milk and/or dairy products with such pathogen due to biofilm formation^7,15^. Moreover, it represents a significant public health issue in hospitals as well as medical care due to the intensified resistance to antimicrobial agents^19,20^.

As a result of upsurge and worldwide antimicrobial resistance, nanomaterials have been used as disinfectants that have proven their efficacy and among these various nanomaterials, copper, silver, and gold^21^. Silver ions and silver-based nano compounds are well known materials that have been used in medicine science 1000 BC^22^. Nano-based silver disinfectants have been proven to have more effective antimicrobial properties due to high surface exposure of the disinfectant to the microbe^23^.

This study aimed to point out the prevalence of biofilm producing *P. aeruginosa* in dairy farm environments passing through different points in milking process and then to evaluate the efficacy of some commonly used disinfectant in the routine disinfection and cleaning regimes in the farm under the study to the efficacy of silver nano-based disinfectants.

## Material and methods

### Study area and period

The current study was performed in a private dairy cattle farm at Beni-Suef locality (coordinates 29° 04ʹ N–31° 05ʹ E), Egypt from December 2019 till September 2020. Dairy cows (*n* = 70) were milked in abreast parlor with two milking machines that were located at the northern part of the farm and near to the yards where the cows were housed on earthy floor that was partially sheltered provided with common water trough and food manager for each group of animals (*n* = 14). The hygienic condition that prevailed in the farm and the dairy was fair.

### Study design

The work in this study was done in two successive steps the first was to investigate the prevalence of biofilm-forming *P*. aeruginosa from different sources by collecting samples such as milk samples, environmental samples besides, human hand swabs that were cultured for the recovery of *P*. *aeruginosa* and their identification was done by molecular identification using *16S rDNA* that is specific for *P*. *aeruginosa* and determination of *toxA* and *fliC* genes, as the most known virulence genes in the identified isolates, then identification of biofilm formation using Congo Red Assay (CRA). The second step was to control this pathogen using dependable disinfectants that are commonly used in the field and loaded disinfectant on sliver nanoparticles, using the broth micro-dilution method. Animal sampling design and protocol was carried out based upon the International Animal Care and Use Committee (IACUC), Ref. No: IORG 0001080), of Beni-Suef University, Egypt, while human samples did not require any approval of Institutional Review Board (IRB) since hand swabs were only collected after oral consent of farm workers. Additionally, the authors confirmed that all methods illustrated in the manuscript were carried out in accordance with relevant guidelines and regulations. All data and results were recorded and statistically analyzed.

### Sample collection

Milk samples (10 ml) (*n* = 50), environmental samples including milk machine, bulk milk tank, water, water trough, feeding manager, feed stuff and flies' samples were screened (*n* = 25 each), and hand swabs (*n* = 25) were obtained from the farm workers under investigation after taking their oral consent, all the workers were apparent healthy at the time of sampling. All samples were collected aseptically according to Munoz et al.^24^ then preserved on ice box to be transported to the lab of Animal Hygiene and Zoonoses on Faculty of Veterinary Medicine, Beni-Suef University, where the bacteriological examination was done. 

### Bacteriological and molecular techniques

Milk samples (one ml each) were pre-enriched into tubes containing 9 ml of the tryptic soy broth while all sample swabs were directly inoculated into tubes containing 9 ml of tryptic soy broth (Oxoid, Basingstoke, UK) and incubated at 37 °C for 24–72 h then a loopful from each tube was inoculated on the surface of freshly prepared cetrimide agar (consisted of peptone, 20 g; Mg chloride, 1.4 g; Potassium sulphate 10 gm; cetrimide 0.3 g; glycerol, 10 ml; agar 13 g per one liter of distilled water). Plates showing large blue–green or green–yellow colonies with characteristic sweet-grape like odor were picked up and biochemically identified^25^. The biochemical tests used to confirm the recovery of *P. aeruginosa* were oxidase, catalase, gelatin hydrolysis and hydrolysis of polysaccharides tests, fermentation of various sugars, pigment production on tryptic soya agar and growth either in 4 °C or 42 °C^26^. The biochemically confirmed isolates were sent to the biotechnology center in the animal health research institute, Egypt for molecular characterization. Where the amplification of *P. aeruginosa* specific *16S rDNA* as well as detection of virulence (*toxA* and *fliC*) genes were performed^27–29^ respectively. Data of primer sequences and target genes were illustrated in Table 1.

**Table 1:** Oligonucleotide and primer sequences specific for *P. aeruginosa* investigated during the study.

Target gene	Primers sequences	Amplified segment (bp)	References
***16S rDNA***	GGGGGATCTTCGGACCTCA	956	Spilker et al.^27^
	TCCTTAGAGTGCCCACCCG		
***Tox A***	GACAACGCCCTCAGCATCACCAGC	396	Matar et al.^28^
	CGCTGGCCCATTCGCTCCAGCGCT		
***Fli C***	TGAACGTGGCTACCAAGAACG	180	Ghadaksaz et al.^29^
	TCTGCAGTTGCTTCACTTCGC		

### Molecular identification

Firstly, DNA was individually extracted from each biochemically confirmed colonies using QIAamp DNA Mini kit (Qiagen, Germany, GmbH). Briefly, 200 µl of the sample suspension was incubated with both 10 µl of proteinase K and 200 µl of lysis buffer at 56 °C for 10 min. After incubation, 200 µl of the absolute ethanol was added up to the lysate. The samples were washed and centrifuged following the manufacturer’s instructions. Lastly, the nucleic acid of each sample was eluted with 100 µl of elution buffer supplied with the kit. For conventional-type PCR amplification, the primers were utilized in a 25 µl volume reaction including 12.5 µl of EmeraldAmp Max PCR Master Mix (Takara, Japan), 1 µl of each primer of 20 pmol concentration, 4.5 µl of water as well as 6 µl of DNA template. The reaction was performed in an Applied biosystem 2720 thermal cycler.

The amplification of *P. aeruginosa* specific *16S rDNA* as well as detection of virulence (*toxA* and *fliC*) genes were performed. The parameter of thermocycling began an initial denaturation cycle at 94 °C for 5 min followed by 30 cycles of the subsequent program, 94 °C for 30 s, the annealing temperatures were 52, 55 and 56.2 °C for 45 s for each primer^27–29^, respectively. The final extension step was at 72 °C for 7 min. The products of PCR were disconnected by electrophoresis on 1.0% agarose gel (Applichem, Germany, GmbH) in 1 × Tris/Borate/EDTA (TBE) buffer at room temperature using gradients of 5 V/cm. For gel evaluation, 40 µl of the PCR products was inserted in each gel slot and the gel was portrayed by a gel documentation system (Alpha Innotech, Biometra) and the data was evaluated using the computer software of DigiDoc-It Imaging System.

### Screening biofilm formation capacity

It was qualitatively investigated using the Congo Red Assay (CRA), by plating on the surface of Congo Red agar (prepared by mixing of brain heart infusion broth (37 g), sucrose (5 g), Congo red dye (0.8 g) and agar (15 g) per a liter of distilled water). Congo red agar plates were inoculated with isolates of *P. aeruginosa*, and incubated aerobically for 24–48 h 37 °C. Following the incubation period, the colonies' color was noticed and interpreted^30^.

### Synthesis and characterization of silver nanoparticle and Virokill silver nanoparticle / composite

The silver colloid was prepared according to Sileikaite et al.^31^ using chemical reduction method through dissolving 0.085 g of silver nitrate in 500 ml distilled water, and then this mixture was heated till boiling. Then 1 g of trisodium citrate was dissolved in 100 ml distilled water and 5 ml of trisodium citrate was added drop by drop to the prepared solution of silver nitrate then it was vigorously mixed. The mixture was then left to heat at 90 °C for 2 h on a hot plate, and then it was left to cool at room temperature. Where the solution color turn into reddish green (end point). The crystal structure and crystallinity of materials were detected using X-ray diffraction (XRD) (Fig. 2), while the vibration and the chemical bonds of the materials were screened by Fourier-transform infrared spectrum (FT-IR) (Fig. 3). The average size and morphological shape of Virokill/AgNPs was characterized by Transmission Electron Microscopy (TEM) (Fig. 4a,b) at the National Research Center (NRC), Egypt.

### Evaluation of germicidal efficacy of tested disinfectants

The germicidal power of two commercially disinfectants proven their efficacy and were approved by food industry^32^; Virokill (potassium per-oxy-mono-sulfate 50.0%, NaCl 3.0%, UBM, Egypt), Peracetic acid (6th October 3rd Industrial Area, Egypt), were tested using different concentration of them against 30 strains of *P.* aeruginosa isolated from milk, human and environmental samples using broth macro-dilution^33^ with different concentrations: Virokill (0.25, 0.5 and 1.0%), peracetic acid (0.25 and 0.5%), after contact times (30 min, 1 h and 24 h).

### Evaluation of germicidal silver nanoparticle and Virokill silver nanoparticle / composite

Different concentrations of silver oxide Ag_2_O NPs (100,150 and 200 mg/L), in addition conc. of 150 and 200 mg/L of Ag NPs were added to 1.0% of Virokill, were tested against 30 traits of *P. aeruginosa* obtained from milk, human and environmental samples using broth macro-dilution method^34^. Ag NPs were added immediately before use and was vigorously shaken using magnetic stirrer to increase the dispersion and prevent settling of NPs over the incubation periods (30 min, 1 h and 24 h).

### Statistical analysis

Data obtained were recorded and the frequency of *P. aeruginosa* in the collected samples as well the germicidal efficacy of tested disinfectants and silver nanoparticles composite were calculated using non-parametric tests (Chi-Square Test) using SPSS (Inc. version 22.0, Chicago, IL, USA).

## Results and discussion

*Pseudomonas* spp. are incontrovertible psy-chrotolerant bacteria that are commonly found in natural environment, such as water, inner side of bulk tanks, cows' teat and surfaces that may associate with the contamination of raw milk^8,35^. Therefore, the searching for probable sources of contamination and adoption of hygienic measures during milking process had become a necessity in the growing dairy industry^18,35^.

The obtained results shown in Table 2 revealed that the total number of positive samples for *P. aeruginosa* isolation were 74 (29.6%) that were recovered from different sources; and this pattern of isolation confirms the ubiquitous nature of the pathogen and variety of media in which it can survive in various conditions^36^.The pathogen was mainly recovered from milk samples (70.0%) followed by swabs of milk machine, hand swabs and milk tank (32.0, 28.0 and 24.0%, respectively) at X^2^ = 156.584 at *P* < 0.001. Much lower results were recorded by Banerjee et al.^36^ who found that the percentage of *Pseudomonas* sp. isolation was only 6.5%, and to some extent Banda et al.^37^ could isolate Gram negative rods (*Pseudomonas* sp.) by the percentage of 10.2%. Conversely to our finding Banda et al.^37^ did not isolate *Pseudomonas* spp. from milk samples but it was isolated (13.3 and 13.0%) from water samples and environmental swabs, respectively. Also, in a study made by Ma et al.^38^ revealed that *P*. *aeruginosa* was detected in the following samples from workers, milk, feed stuff, floor surface and water samples by (1.0, 10.0, 4.0, 4.0 and 6.0%, respectively). Similarly, Vidal et al.^39^ mentioned that they were able to isolate *Pseudomonas* spp. from teat cups of milking machine and bulk tank (95.0%), milk samples (90.0%), from milkers' hands (80.0%), teat surface (50.0%), and water sample (70.0%).

**Table 2:** Prevalence of *P. aeruginosa* recovered from animals, their environment, and humans during the study period.

Samples/swabs	Examined samples (No.)	Positive samples (No.)	Prevalence of *P. aeruginosa* (%)
Milk	50	35	70.0
Hand swab	25	7	28.0
Milk tank	25	6	24.0
Milk machine swab	25	8	32.0
water	25	3	12.0
Water trough swab	25	4	16.0
Feed stuff	25	3	12.0
Feed manager swab	25	4	16.0
flies	25	4	16.0
Total X2 = 156.584 P. value < 0.001	250	74	29.6

Concerning the prevalence of biofilm producing capacity of isolated *P. aeruginosa* collected from different samples (Table 3) it showed that out of 74 recovered *P. aeruginosa* traits, 50 (67.5%) were biofilm producers meanwhile 24 (32.4%) were non-biofilm producers, and this prove a significant increase in the biofilm propriety of the isolated pathogen at X^2^ = 30.757 at *P* < 0.001, referring to their distribution these isolates were mainly obtained from environmental samples followed by milk and finally from human samples (23 (71.8%), 22 (62.8%), 5 (71.4%). Biofilm is a microbial property that is characterized by adhesion of the microbes to a solid surface and production of a matrix that surrounds and includes the bacterial cells and include extracellular polysaccharides, proteins, and DNA^40–43^ proved that biofilm protects bacteria from the most sever adverse environmental conditions including antimicrobials. Banda et al.^37^ recorded lower results to that found in this study where he recorded that only 14 out of 86 (16.3%) of microbial isolates were strong biofilm formers and (20.9%) did not form any biofilm. Also, Milivojevic et al.^44^ reported that only30 out of 202 isolates (15.0%) were biofilm former and 11 (5.0%) were non biofilm former. Meanwhile Ngo et al.^45^ recorded much higher results than those reported in the following study where he found that (100.0%) of tested isolates were biofilm former with different degree ranging from strong, moderate, or weak.

**Table 3:** Prevalence of the biofilm producing capacity of isolated *P. aeruginosa* collected from samples during the study period.

Samples/swabs	Examined samples (No.)	Biofilm-procedures	Non-biofilm procedures
Milk	35	22 (62.8%)	13 (37.1%)
Hand swab	7	5 (71.4%)	2 (28.5%)
Environment	32	23 (71.8%)	9 (28.1%)
Total X2 = 30.757 P > 0.001	74	50 (67.5%)	24 (32.4%)

**Figure 1:**
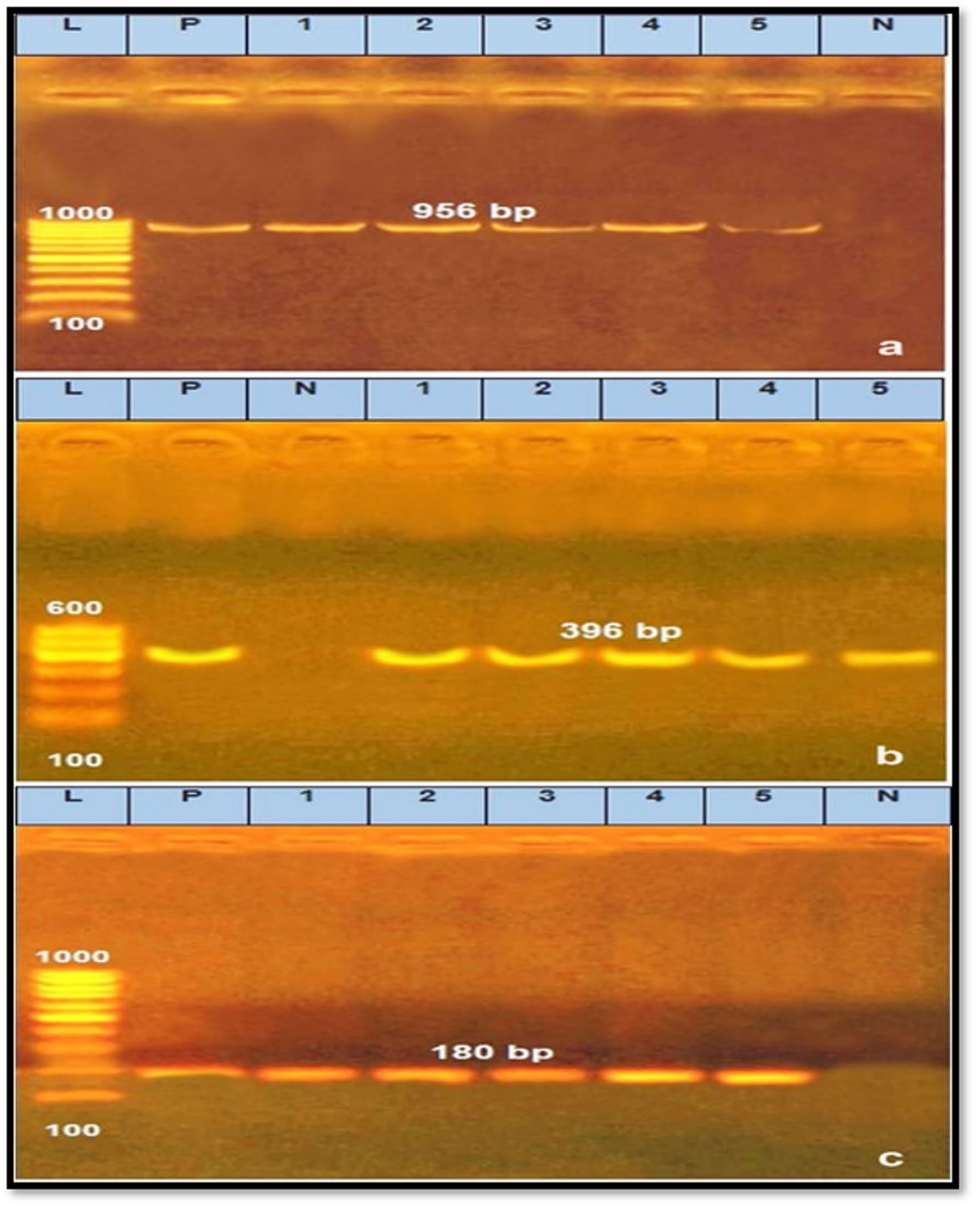
Agarose gel electrophoresis for PCR products of *P. aeruginosa* species identification using *16S rDNA* gene (**a**), amplified 356 bp and the virulence genes (**b**,**c**), amplified 396 and 180 bp for *toxA* and *fliC* genes, respectively. Lane (L): 100 bp Ladder ‘’Marker’’, Lane): 1–5), the examined samples, Lane Pos: Positive control, Lane Neg: Negative control.

Figure 1 referring to the distribution of both *tox* and *fliC* genes; showed that all the randomly selected isolates were caring both genes. *fliC* (flagellin) has an important role in stimulating the immune response, with the emergence of multi-resistant strains^46,47^. The virulence gene exotoxin A (*tox A*) is secreted through Type II secretion mechanism, which secrete proteins into the extracellular environment, including lipase, phospholipase, alkaline phosphatase, and protease^48,49^. These results are in accordance with the results of biofilm formation of the tested isolates that also assist these pathogens to resist antimicrobial agents (both disinfectants and antibiotics).

**Figure 2:**
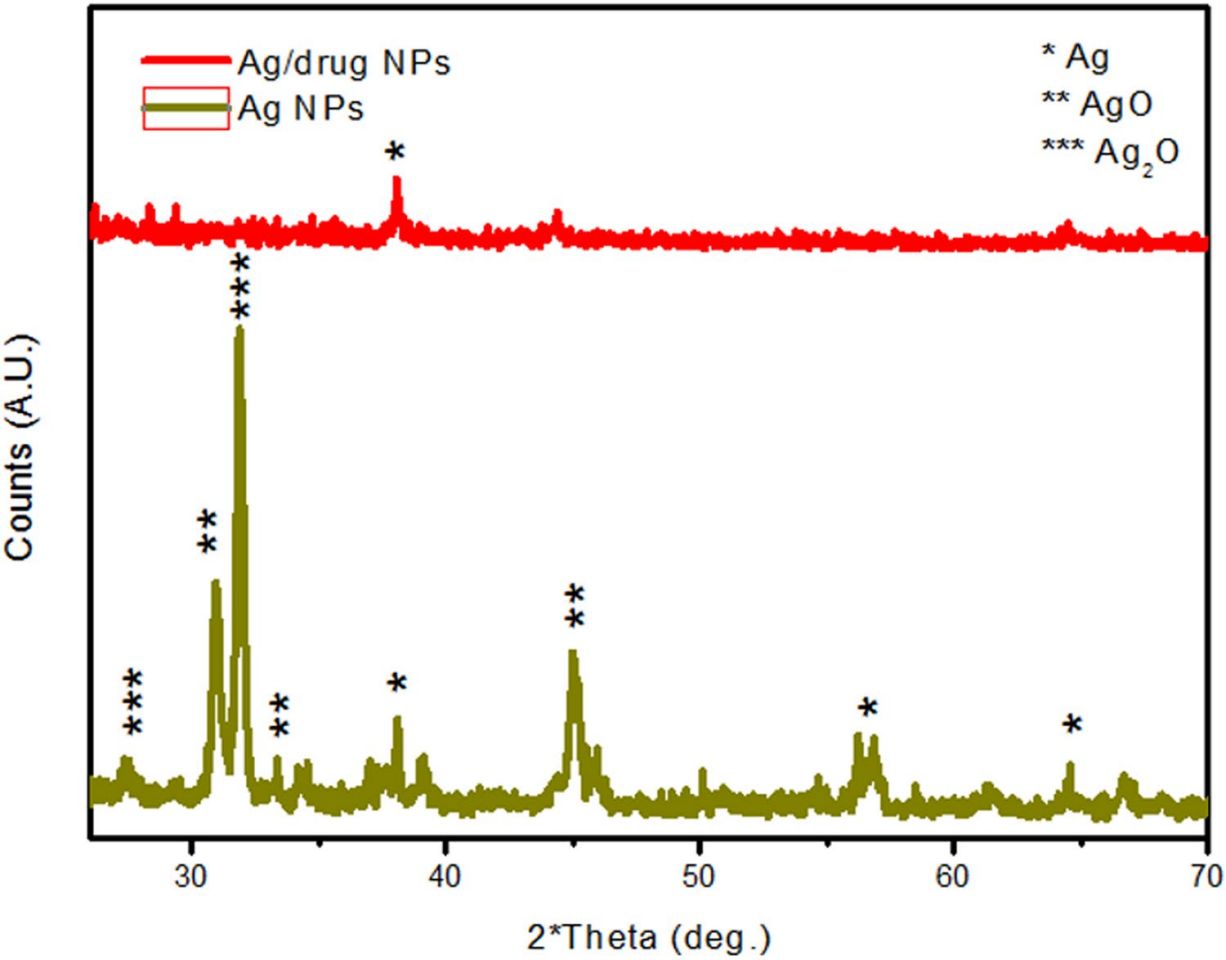
X-ray Diffraction (XRD) of silver nanoparticles before and after Virokill loading, showing characteristic peaks for silver and its oxides AgO and Ag_2_O, indicating successfully incorporated of Virokill disinfectant within the silver nanoparticles.

Figure 2 shows the XRD analysis of silver nanoparticles before and after drug Virokill loading. As shown, characteristic peaks for silver and its oxides AgO and Ag_2_O appeared in the diffractogram. The peaks at 38, 56.1 and 64.4 could be attributed to the diffraction planes of (111), (142) and (220) of silver respectively^50,51^. Peaks at 27.4 and 31.9 corresponds to (110) and (111) planes of Ag_2_O, respectively^49^. While peaks at 30.9, 33.3 and 45 corresponds to (111), (202) and (132) planes of AgO, respectively^52,53^.

**Figure 3:**
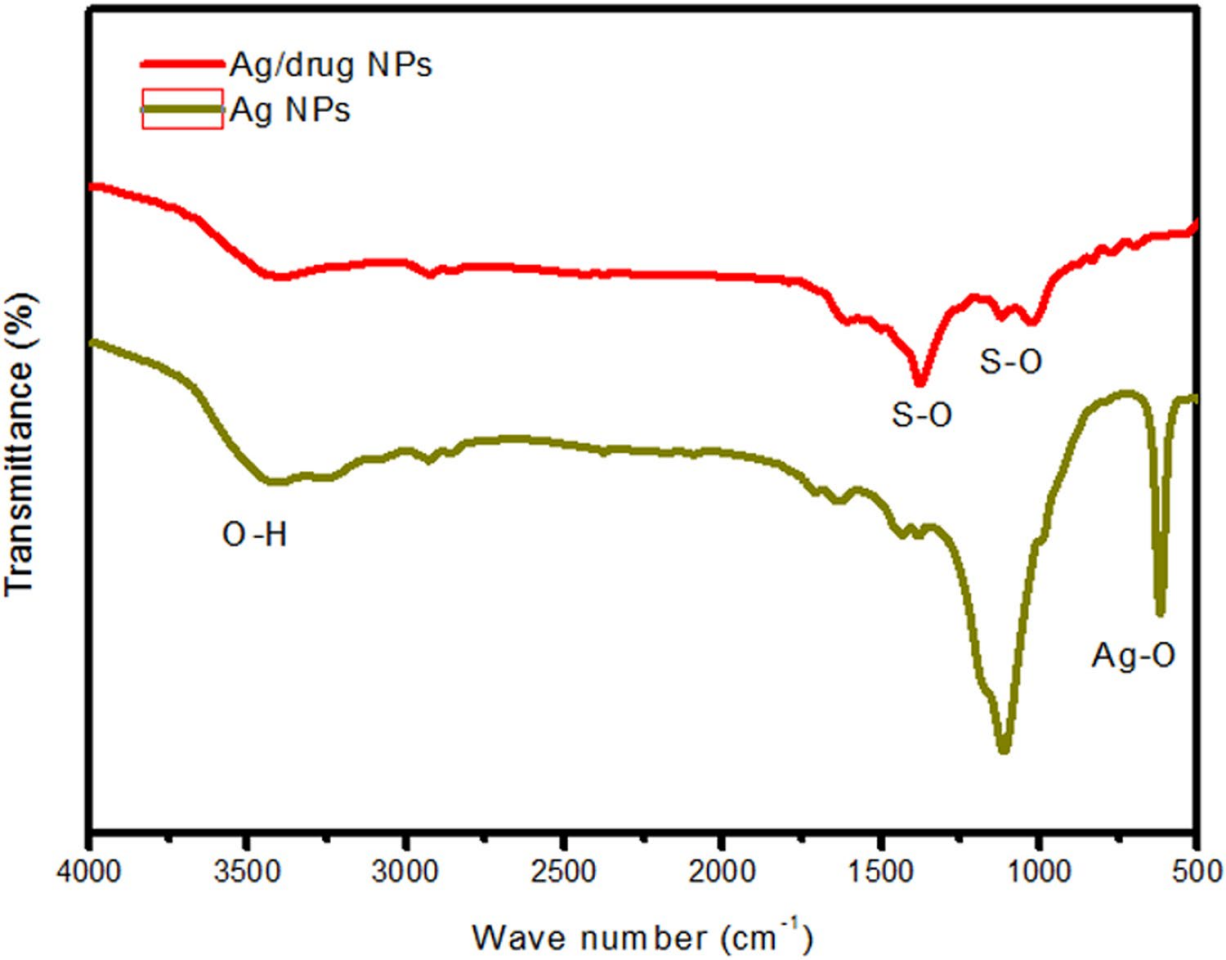
Fourier-transform infrared spectra (FTIR) of silver nanoparticles before and after Virokill disinfectant loading.

Figure 3 shows the FTIR spectra of the prepared silver nanoparticles. The broad band around 3400 cm^−1^ can be assigned to the stretching of O–H bond in adsorbed water molecules. The low intensity peak at 600 cm^−1^ originates from the Ag–O bond^54^. After drug Virokoll loading, the characteristic peaks form S–O stretching vibration for SO_4_^2−^ and HSO_5_^−^ in the per-oxy-mono-sulfate appeared at approximately 1440 cm^−1^ and 1116 cm^−1^, respectively^55^.

**Figure 4:**
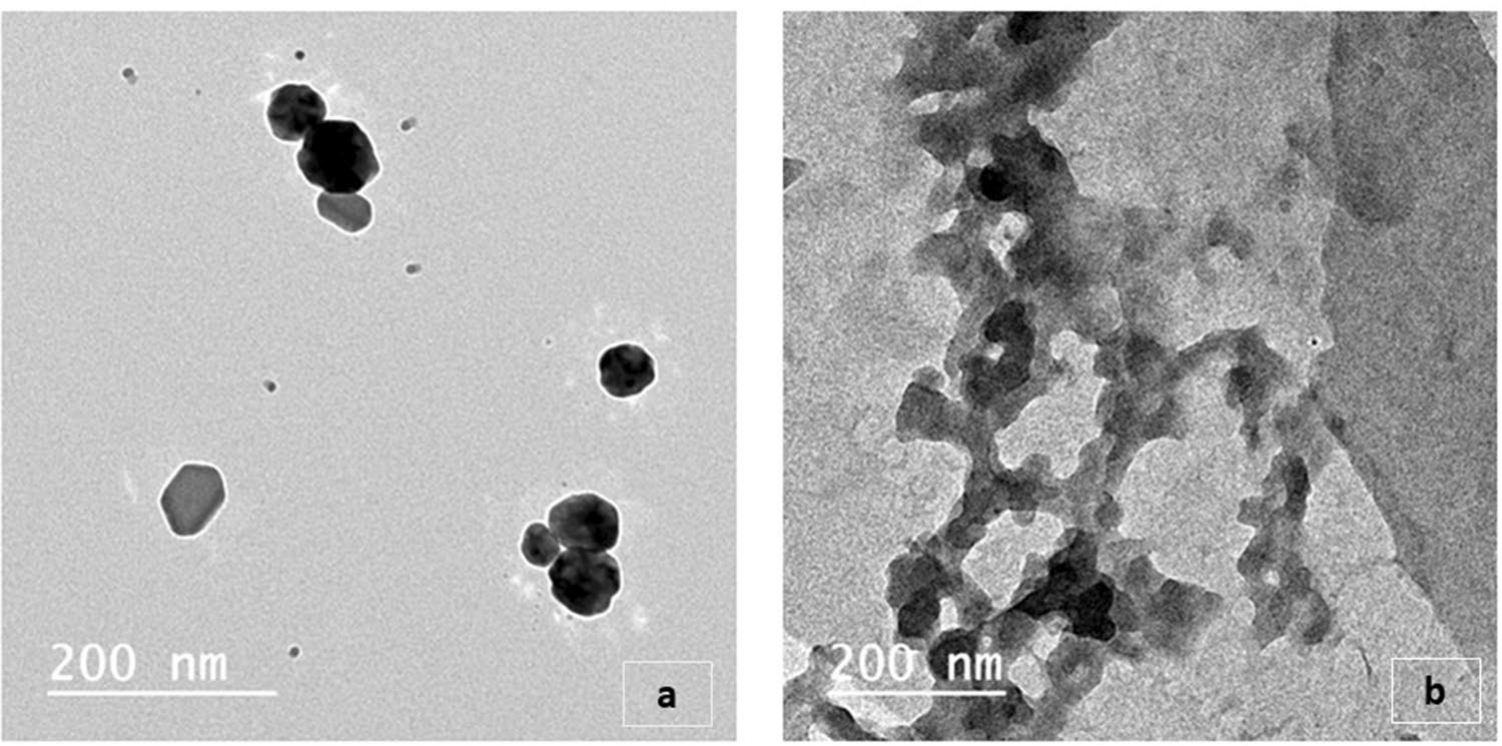
Transmission electron microscopy (TEM) images of silver nanoparticles before loading, showing a spherical like shape with diameters ranging amongst 36–60 nm (**a**) while in after loading (**b**), appears that the Virokill is completely encapsulates the silver nanoparticles.

Referring to Transmission electron microscopy (TEM) image (Fig. 4a of silver nanoparticles it shows that the particles have a spherical like shape with diameters ranging between 36–60 nm. After the Virokill loading, the TEM image (Fig. 4b) shows that the Virokill completely encapsulates the silver nanoparticles Ag NPs within its matrix.

Results illustrated in Table 4 showed that the sensitivity of *P. aeruginosa* was significantly high to Virokill/AgNPs (200/1000) mg/L after 24 h followed by Ag/NPs (200/1000) mg/l after 24 h at P < 0.001 (70.0 and 55.0%, respectively). On the other hand, it showed a significant high degree of resistance to Peracetic acid 0.25% and 0.5% after 30 min and 1 h of exposure (90.0, 75.0% and 80.0, 65.0%, respectively). Generally, the pathogen showed a variable degree of resistance to most of the used disinfectants at different contact times and concentrations, yet this resistance decreases with the increase of both concentration and exposure time of this disinfectant. Despite the common use of these disinfectants in dairy farms from awhile, and even so these results might be shocking but not actually surprising which might be referred due to the insufficient cleaning of the farm and removal of organic matter and/or the misuse of disinfectants (following the label instructions)^56,57^. Eventually that have led to increase resistance of food borne pathogens to biocides^58^ with the probability of cross transfer of genes that responsible for their resistance to both disinfectants and antibiotics in the environment this became of great interest to researchers everywhere to overcome and/or to prevent this from happening so saving money and protecting humans' life^59–61^. The significant high degree of bacterial resistance recorded in this study might be attributed to the ability of these pathogens to form biofilm and owning virulence genes which have been proved during the study, also might be due the modest hygienic practices followed in the farm under the study, where presence of organic matter suggests the establishment of biofilm formation on the corresponding surfaces^40^ and improper use of disinfectants according to the manufacturers' instructions^58^. On the contrary to our results Gibson et al.^62^ proved that the use of acids such peracetic acid significantly (*P* < 0.05) affected the viability of *S. aureus* and *P. aeruginosa* and it was not necessary to use another disinfectant for cleaning. Stewart et al.^63^ reported that chloramines (chlorosulfates as Virokill) were able to penetrate biofilm 6–8 times sooner than free chlorine however these bacteria were highly resistant to both agents. Ag/NPs were proven to damage the bacterial cell membranes and finally their death^64^. Moreover, silver nanoparticles are known for their lethal effect to different bacterial pathogens^65^ with special reference to the shape and size of nanoparticles^65^ that was detected by TEM in this study. Since Virokill showed better results than per-acetic acid it was loaded on Ag/NPs to improve its penetration into the bacterial cells particularly in biofilm and Ag/NPs acts in synergism together with the disinfectant to improve its biocidal efficiency^66^.

**Table 4:** The bactericidal efficacy of tested disinfectants, silver nanoparticles and its loaded forms against *P. aeruginosa* traits.

Tested disinfectant/bacteria (no.)	Conc (mg/L)	Sensitivity pattern (%) of *P. aeruginosa* isolates at different exposure times								
		30 min			1 h			24 h		
		S	I	R	S	I	R	S	I	R
Peracteic acid^®^	0.25%	10.0	0.0	90.0	15.0	5	80	30.0	20.0	50.0
	0.5%	20.0	5	75.0	25.0	10	65	35.0	25.0	40.0
Virokill^®^	0.25	15.0	10	75.0	20.0	15	65	25.0	20.0	55.0
	0.5	20.0	10	70.0	25.0	20	55	30.0	30.0	40.0
	1.0%	35.0	0.0	65.0	30.0	10	60	45.0	10.0	45.0
AgNPs	150	15.0	10	75.0	15.0	10	75	20.0	15.0	65.0
	200	20.0	15	65.0	30.0	10	60	55.0	15.0	30.0
Virokill/AgNPs	150	25.0	5	70.0	30.0	20	50	40.0	20.0	40.0
	200	30.0	10	60.0	35.0	25.0	40.0	70.0	20.0	10.0
P value	0.001				0.001			0.001		

*S* Sensitive, *I* Intermediate, *R* Resistant.

## Conclusion

From the current study it was concluded that *P. aeruginosa* prevailed in dairy farm environment and the more there is a lack of hygiene the more their ability to form biofilm will increase and eventually become more virulent and resistant to both disinfectants and antibiotics. The increased resistance to antimicrobial agents possesses a risk to humans' and animals' health through the infection of dairy animals' and contamination of dairy products. Therefore, we recommend to design and activate a routine cleaning and disinfection program that initially clean and remove organic matter which protects these pathogens and hinder the efficacy of the used disinfectant starting from the animal house and passing through the entire system of dairy production. Using Virokill/AgNPs composite (200/1000) mg/L that was proven its efficacy and allowing it to act on the pathogen for 24 h to maintain clean and healthy dairy environment, increase the milk quality and improve the dairy industry.
